# Early Hyperprogression of Rhabdomyosarcoma Detected by ^18^ F-FDG PET/CT Three Weeks after CAR-T Treatment

**DOI:** 10.1055/s-0044-1787537

**Published:** 2024-06-20

**Authors:** Shenrui Guo, Zhen Tan, Wenbin Guan, Yafu Yin

**Affiliations:** 1 Department of Nuclear Medicine, Xinhua Hospital, Shanghai Jiao Tong University School of Medicine, Shanghai, China; 2 Department of Pediatric Hematology/Oncology, Xinhua Hospital, Shanghai Jiao Tong University School of Medicine, Shanghai, China; 3 Department of Pathology, Xinhua Hospital, Shanghai Jiao Tong University School of Medicine, Shanghai, China

**Keywords:** ^18^ F-FDG PET/CT, hyperprogression, rhabdomyosarcoma, CAR-T treatment, pediatric

## Abstract

Chimeric antigen receptor T-cell (CAR-T) treatment has been widely used in the treatment of hematological malignancies, and its application has been gradually expanded to the research and treatment of solid tumors. However, unconventional types of response may occur after CAR-T treatment, such as hyperprogression, resulting in terrible outcomes. Here, we report the case of a 13-year-old adolescent boy with relapsed and refractory rhabdomyosarcoma who developed early hyperprogression 3 weeks after CAR-T treatment (target: B7H3 and CD171), which was detected by fluorine-18 fluorodeoxyglucose ( ^18^ F-FDG) positron emission tomography (PET)/computed tomography (CT). The patient eventually underwent amputation. Attention should be paid to the possibility of early hyperprogression after CAR-T treatment, and ^18^ F-FDG PET/CT has an absolute advantage in early evaluating treatment response to immunotherapy.

## Introduction

Rhabdomyosarcoma (RMS) is the most common soft-tissue sarcoma in children and adolescents, accounting for 3 to 4% of all pediatric cancers. 1 There are different treatments for RMS, such as surgery, radiotherapy, and chemotherapy. Immunotherapy, including chimeric antigen receptor T-cell (CAR-T) treatment, has been widely used in the treatment of hematological malignancies, and it also has significant clinical benefits in solid tumors such as RMS. It is reported that a 7-year-old boy with metastatic RMS has achieved durable remission after CAR-T treatment. 2 However, several special responses were observed after checkpoint blockade. One is hyperprogression, a phenomenon reflecting an extraordinarily rapid tumor progression following immunotherapy, which is detrimental. 3 Here, we report a case of early hyperprogression 3 weeks after CAR-T treatment detected by fluorine-18 fluorodeoxyglucose ( ^18^ F-FDG) positron emission tomography (PET)/computed tomography (CT) in a 13-year-old adolescent boy with relapsed and refractory RMS.

## Case Report

**Fig. 1:**
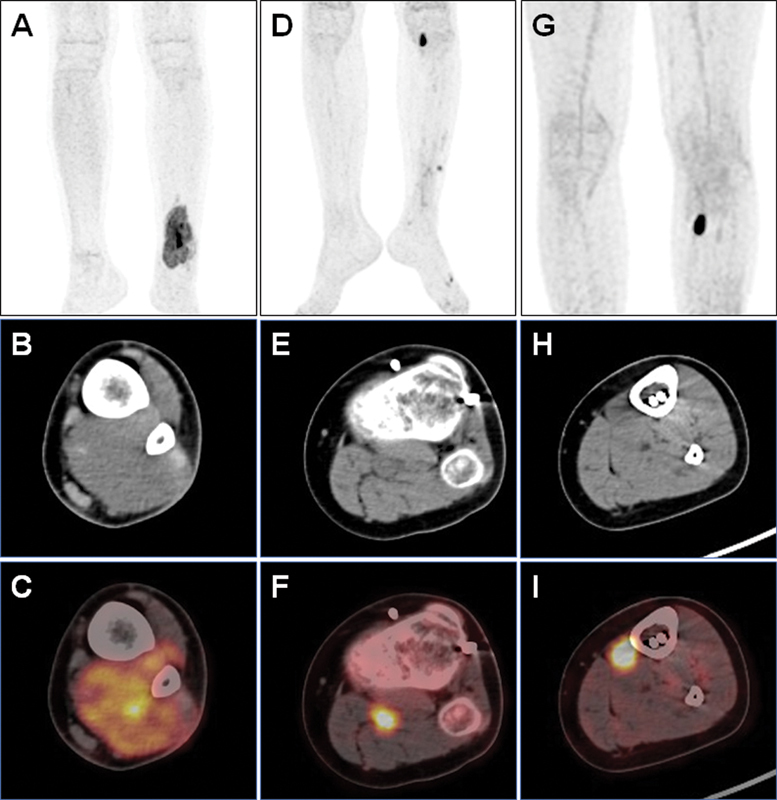
Fluorine-18 fluorodeoxyglucose ( ^18^ F-FDG) positron emission tomography (PET)/computed tomography (CT) imaging ( **A, D, G** : maximum intensity projection [MIP] image; **B, E, H** : axial CT image; **C, F, I** : axial PET/CT fused image). ( **A–C** ) The first PET/CT scanning showed that there was an uneven soft-tissue mass in the muscle of the distal left lower limb, with a volume of about 96 mm × 45 mm × 37 mm, and the FDG uptake was abnormally increased (SUVmax: 10.4). ( **D–F** ) The fourth PET/CT scanning suggested a relapse in the left popliteal fossa, a soft-tissue nodule with increased FDG uptake (SUVmax: 10.1). ( **G–I** ) The fifth scanning showed the third relapse, a soft-tissue nodule with increased FDG uptake (SUVmax: 13.7) near the tibia in the proximal medial side of the left lower limb (about 4 cm below the knee).

In March 2020, a 13-year-old adolescent boy was admitted to the hospital with a lump in his left lateral ankle. Biopsy showed alveolar RMS with PAX3-FOXO1 gene fusion. ^18^ F-FDG PET/CT ( Fig. 1A–C ) showed an uneven soft-tissue mass in the distal muscle of the left lower limb, with abnormal increased FDG uptake (maximum standard uptake value [SUVmax]: 10.4). The patient received four cycles of preoperative chemotherapy (Chinese Children Cancer Group-RMS-2016 [CCCG-RMS-2016], phase III, medium-risk group) and then underwent surgery on July 27, 2020, followed by 14 cycles of chemotherapy and radiotherapy. No obvious abnormality was found in PET/CT images (on September 15, 2020 and September 17, 2021) during follow-up.

Later, the patient experienced two relapses in the lateral left lower limb (January 2022) and left popliteal fossa (June 2022; Fig. 1D–F ), both of which were confirmed by surgical resection and biopsy. On November 24, 2022, the patient received autologous peripheral blood hematopoietic stem cell transplantation, and 2.98 × 10 ^6^ CD34 positive selected peripheral blood stem cells per kilogram body weight were reinfused (603 mL, with 91% cell activity). However, in April 2023, RMS relapsed on the proximal medial of the left lower limb ( Fig. 1G–I ).

**Fig. 2:**
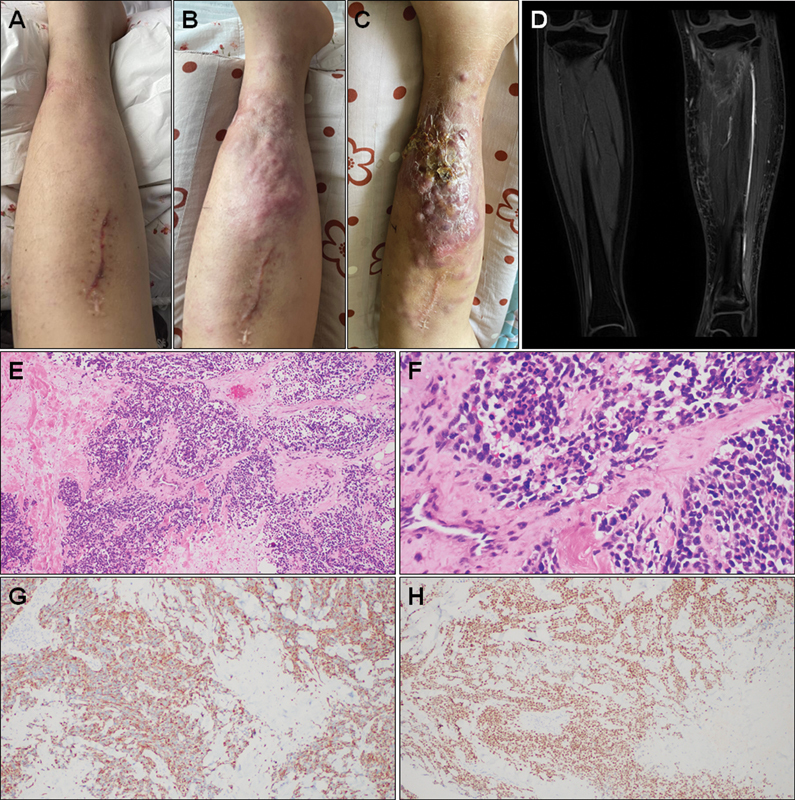
( **A–C** ) Progress of skin redness, swelling and ulcers of left lower limb. ( **D** ) Magnetic resonance imaging (MRI) showed several flaky abnormal signals in the left tibia, abnormal enhancement of soft tissue around the upper and lower tibia, and edema of soft tissues on the left lower limb (May 23, 2023). ( **E** ) The tumor cells were arranged in nests and sheets (hematoxylin and eosin, H&E; 100×). ( **F** ) The tumor cells have obvious atypia, abundant and acidophilic cytoplasm, nuclear deviation, and obvious mitosis and apoptosis (H&E, 400×). ( **G, H** ) Immunohistochemistry shows DES (+) and MYOD1 (+) of the tumor tissue.

**Fig. 3:**
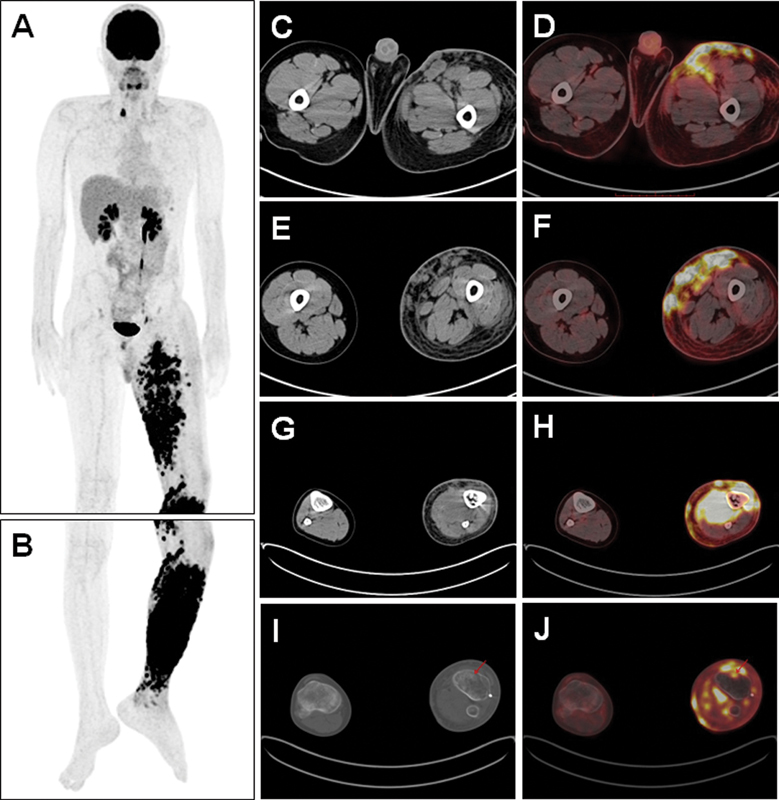
( **A, B** ) The sixth positron emission tomography (PET)/computed tomography (CT) showed new lesions in the left lower limb 2 months after chimeric antigen receptor T-cell (CAR-T) treatment (maximum intensity projection [MIP] image). ( **C, E, G, I** ) Axial CT images and ( **D, F, H, J** ) axial PET/CT fusion images showed obvious subcutaneous edema in the left lower limb, and solid nodules with increased fluorodeoxyglucose (FDG) uptake (SUVmax: 28.6) in the skin, subcutaneous tissues, and muscles in the left lower limb.

After the operation for recurrent lesion, the patient received CAR-T treatment (target: B7H3 and CD171) on April 28, 2023, and developed fever the next day. Because cytokines were normal, his fever was thought to be caused by immune response. On May 17, 2023, the patient developed fever, redness and swelling of the left lower limb, rising skin temperature and ulceration ( Fig. 2A–D ), and no pain or other discomfort. Routine clinical biochemistry showed an increased level of C-reactive protein (146 mg/L). Anti-infection treatment (e.g., vancomycin, fluconazole, doxycycline) achieved little effect, while slight relief occurred after chemotherapy (cyclophosphamide 400 mg/m ^2^ days 1–5 + topoisomerase 1.2 mg/m ^2^ days 1–5). Later, the sixth PET/CT scanning ( Fig. 3 ) was performed on July 10, 2023, which showed there was obvious subcutaneous edema in the left lower limb, and countless solid nodules with increased FDG uptake (SUVmax: 28.6) were visible in the muscles, subcutaneous tissues, and skin of the left lower limb. It was considered to be recurrence of RMS, invading extensive subcutaneous, muscles and skin in the left lower limb. At the same time, the possibility of coinfections could not be ruled out. Then ultrasound-guided puncture biopsy was performed, and it confirmed the recurrence of alveolar RMS ( Fig. 2E–H ). The patient developed hyperprogression, and finally underwent amputation. The postoperative pathology suggested that the tumor was distributed in a multinodular pattern, with partial necrosis, extending to the subcutaneous and dermal layers, and partially invading the epidermis, and tumor thrombi could be seen in local veins.

## Discussion

CAR-T treatment is a revolutionary new pillar in cancer treatment. 4 In addition to the exhilarating clinical efficacy in the treatment of hematological malignancies, CAR-T treatment also shows clinical benefits in solid tumors, including glioblastoma, neuroblastoma, sarcoma, and pancreatic cancer. 5 Hegde and his colleagues also reported a 7-year-old boy with metastatic RMS who achieved sustained relief after receiving two stages of CAR-T treatment, despite bone marrow relapse 6 months after the end of the first stage of CAR-T treatment. 2 Unfortunately, some patients may experience hyperprogression of disease after CAR-T treatment. Hyperprogression is an extraordinary response to immunotherapy including CAR-T treatment, which is characterized by accelerated disease progression and worsening survival outcomes. 3 6 7 8 It is reported that the incidence of hyperprogression ranges from 4 to 29%, 6 which may occur in various tumors, such as lymphoma, 9 non-small-cell lung cancer, 10 hepatocellular carcinoma, 11 12 renal cell carcinoma, 13 melanoma, 14 breast cancer, 15 sinonasal cancer, 16 17 bladder carcinoma, 18 and gastrointestinal cancer. 19 However, limited literature is available regarding hyperprogression in RMS. Here, we report a pediatric patient with relapsed and refractory RMS who developed early hyperprogression 3 weeks after CAR-T treatment. This suggests that such rapid hyperprogression may occur after CAR-T treatment, and we need to be aware of the unusual tumor response to immunotherapy.

However, the potential mechanism of hyperprogression remains unclear. The predictive markers of hyperprogression also need further exploration. Several factors, such as the phenotype of CD8 and CD4 T cells, MDM2/MDM4 amplification, EGFR alterations, old age, high metastatic burden, and locoregional recurrences in the radiation field may be related to hyperprogression. 6 Early and accurate identification of hyperprogression is crucial, but also difficult. Considering the differences in clinical outcomes, it is very important to distinguish between hyperprogression and pseudoprogression, which is characterized by infiltration of inflammatory cells in tumor sites rather than tumor cells. 6 In addition to biopsy, medical imaging could also help identify these responses with the help of new technologies. 8 In this case, ^18^ F-FDG PET/CT is helpful to identify hyperprogression from inflammatory response. ^18^ F-FDG PET/CT plays an important role in evaluating treatment response to immunotherapy at different time points during therapy, especially in early evaluation, so as to guide the therapeutic strategies. 20 21 22 A series of PET imaging in short intervals might be important to identify hyperprogression. 23 This case indicates that early detection of PET/CT may be more valuable for evaluating the response to CAR-T treatment, and it is necessary to consider early detection of PET/CT as an important evaluation tool after CAR-T treatment.

## Conclusion

Hyperprogression is an unconventional but not rare response to immunotherapy. It should be noted that rapid hyperprogression may occur after CAR-T treatment. It is important to identify hyperprogression early in order to avoid detrimental outcomes, and performing ^18^ F-FDG PET/CT as soon as possible may be helpful.
